
JOURNAL INFORMATION
==============================
NLM Title Abbreviation: ScientificWorldJournal
Journal ID: ScientificWorldJournal
NLM Journal ID: 101131163
Journal ID: TSWJ

The Scientific World Journal

ISSN: 2356-6140
EISSN: 1537-744X
Publisher: Wiley

ARTICLE INFORMATION
==============================
PMCID: PMC6083845
PMID: 30147621
DOI: 10.1100/tsw.2001.245
Article ID: 129296
Article version: 1
Subjects: Short Report

PAK-2 Targeting to Mitochondria and Cleavage by Caspases Induces Apoptosis

Rudel Thomas *
Laumlttig Kathrin
Machuy Nikolaus
Manke Tabita
Muumuller Anne
Thieck Oliver
Max-Planck-Institute for Infection Biology, Dept. of Molecular Biology, Schumannstr. 21/22, 10117 Berlin, Germany
*Thomas Rudel: rudel@mpiib-berlin.mpg.de
Publication date: 2001
Electronic publication date: 2001 Sep 24
Volume: 1
First page: 144
Last page: 144
Copyright: Copyright © 2001 Thomas Rudel et al.
Copyright year: 2001
License: This is an open access article distributed under the Creative Commons Attribution License, which permits unrestricted use, distribution, and reproduction in any medium, provided the original work is properly cited.
License URL: https://creativecommons.org/licenses/by/3.0/

Funding: Deutsche Forschungsgemeinschaft

==============================
Miami *Nature Biotechnology* Short Reports

*TheScientificWorld* (2001) 1 (S3), 144SR

ISSN 1532-2246; DOI 10.1100/tsw.2001.245

### PAK-2 TARGETING TO MITOCHONDRIA AND CLEAVAGE BY CASPASES INDUCES APOPTOSIS

Thomas Rudel*, Kathrin Lättig, Nikolaus Machuy, Tabita Manke, Anne Müller and Oliver Thieck

Max-Planck-Institute for Infection Biology, Dept. of Molecular Biology, Schumannstr. 21/22, 10117 Berlin, Germany
* rudel@mpiib-berlin.mpg.de

**INTRODUCTION.** p21-activated kinase-2 (PAK-2), but not the highly homologous PAK-1, is cleaved and activated by caspases during apoptosis (1). The caspase cleavage product of PAK-2 efficiently induces apoptosis (2) while constitutive active PAK-1 has been implicated to play a role in survival pathways (3,4). We have analyzed the molecular basis of the opposite function elicited by PAK-1 and PAK-2.

**METHODS.** cDNAs encoding wild type (WT) or mutant PAK-1 or PAK-2 were transfected into different cell lines. Mitochondrial localization was monitored by biochemical methods and by confocal microscopy. WT and mutant PAKs were artificially targeted to cellular compartments by the addition of targeting signals to the N- or C-terminus. Apoptosis was monitored by microscopy and immunostaining of cytochrome c or active caspase-3.

**RESULTS.** Here we demonstrate that cleavage of PAK-2 by caspases results in translocation of the active kinase to mitochondria and induction of apoptosis. Translocation depends on the kinase function because a kinase-deficient derivative of PAK-2 was not found in mitochondria. Translocation to the mitochondria is an absolute prerequisite for PAK-2 to elicit a pro-apoptotic effect since artificial targeting of the kinase fragment to the cytoplasmic membrane abrogated the induction of apoptosis. PAK-2 artificially transported to the mitochondrial inter-membrane space induced the release of cytochrome C. Cytochrome C release induced by PAK-2 *in vivo* occurs independently of caspase function, but was efficiently blocked by Bcl-2. Interestingly, targeting of a dominant-negative PAK-2 kinase fragment to mitochondria suppressed apoptotic phenotypes induced by death receptors. Our data suggest the direct involvement of the PAK-2 kinase in apoptotic signaling from death receptors to mitochondria.

**DISCUSSION.** We have shown that the highly homologues PAK-1 and PAK-2 kinases localize to different cellular compartments. Since only the PAK-2 kinase, but not the PAK-1 kinase, targeted mitochondria, the different localization is most likely the reason for their function in apoptosis and cell survival or proliferation, respectively. This model is fully supported by the results obtained by artificially targeting the PAK kinases to different cellular compartment. The inhibition of TNF-induced apoptosis by dominant-negative PAK-2 targeted to the mitochondrial inter membrane space suggested that possible substrates or interaction partners might reside in this compartment. Future work will concentrate on identifying them and defining their role in inducing apoptosis via mitochondria.

**ACKNOWLEDGEMENT.** This work was supported by the Deutsche Forschungsgemeinschaft (DFG).

**REFERENCES.**

1. Rudel, T. and Bokoch, G.M. (1997) Science 276, 1571-1574
2. Rudel, T., Zenke, F.T., Chuang, T.H., and Bokoch, G.M. (1998) J. Immunol. 160, 7-11
3. Mira, J.P., Benard, V., Groffen, J., Sanders, L.C., and Knaus, U.G. (2000) Proc. Natl. Acad. Sci. U S A 97, 185-189
4. Schurmann, A., Mooney, A.F., Sanders, L.C., Sells, M.A., Wang, H.G., Reed, J.C., and Bokoch, G.M. (2000) Mol. Cell. Biol. 20, 453-461
